# New COVID-related results for estimating the shadow economy in the global economy in 2021 and 2022

**DOI:** 10.1007/s10368-022-00537-6

**Published:** 2022-07-05

**Authors:** Friedrich Schneider

**Affiliations:** Research Institute of Banking and Finance, Johannes Kepler University, Altenbergerst. 69, 4040 Linz, Austria

**Keywords:** Shadow economy, OECD, Europe, Effect of the Coronavirus pandemic, Latest shadow economy results for 2021 and 2022, C39, C51, C82, H11, H26, U17

## Abstract

Considering the development of the shadow economy of 36 European and OECD countries over the period from 2003 to 2022 and the effect of the Coronavirus pandemic from 2020 onwards, the average size of the shadow economy of 36 European and OECD countries decreased from 16.48% of GDP in 2020 to 16.07% in 2021 (a decline of 0.41 percentage points). Due to a continued (forecasted) economic recovery in 2022, the average shadow economy of these 36 countries will slightly increase to 15.96% of GDP (average of all 36 countries): a very modest reduction of 0.11 percentage points.

## Introduction: a short explanation how to estimate a shadow economy

In this introductory section, I begin by making some short remarks on the question of how to estimate a shadow economy (SE).^1^ The most often used methodology to estimate the size of the shadow economy is based on a combination of the cash (currency demand) approach and of the Multiple Indicators and Multiple Causes (MIMIC) method. The basic idea behind the currency demand approach is that goods and services sold in the shadow economy are paid for in cash and that, using a cash demand function, it is possible to estimate such goods and services provided and performed, respectively, in return for cash and thus to calculate the volume (value added) of the shadow economy. The MIMIC approach is based on the idea that the size of the shadow economy is not a directly observable figure, but that it is possible to approximate it using quantitatively measurable causes of working in the underground economy (such as the tax burden and the amount of regulation) and using indicators (such as cash and the official labor force participation rate), in which shadow economic activities are reflected. As the MIMIC method only enables relative orders of magnitude of the underground economy of individual countries to be calculated, some SE values calculated with the help of the cash approach are necessary to convert/calibrate the SE values into absolute ones (i.e., in percentage of official GDP or in billions of Euros).

**Fig. 1:**
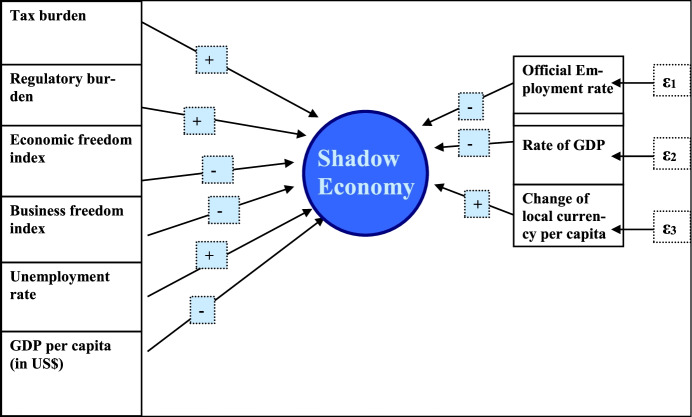
MIMIC estimation procedure. Source: Medina and Schneider (2018)

In the following, the MIMIC estimation procedure (see also Fig. 1) is briefly explained^2^:

1. Modeling the shadow economy as an unobservable (latent) variable
2. Description of the relationships between the latent variable and its causes in a structural model:
3. The link between the latent variable and its indicators is represented in the measurement model:  .

where:

*η*: latent variable (shadow economy).
*X*: (q × 1) vector of causes in the structural model.
*Y*: (p × 1) vector of indicators in the measurement model.
*Γ*: (1 × q) coefficient matrix of the causes in the structural equation;
*Λy*: (p × 1) coefficient matrix in the measurement model;
*ζ*: error term in the structural model and ε is a (p × 1) vector of measurement error in y.

The specification of the structural equation is: 
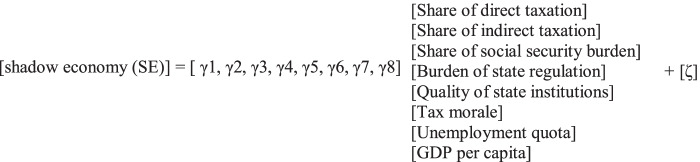


The specification of the measurement equation is:where *γi* and *λi* are coefficients to be estimated.

**Table:** 

Employment quota		λ1				ε1
Change of local currency	=	λ2	**x**	**Shadow economy**	+	ε2
Average working time		λ3				ε3

How does one proceed to get the absolute figures? Feld and Schneider (2010) use the following two steps:

1. The first step is that the shadow economy remains an unobserved phenomenon (latent variable) which is estimated using the causes of illicit behavior, e.g., the tax burden and regulation intensity, and indicators reflecting illicit activities, e.g., currency demand and official work time. This procedure “produces” only relative estimates of the size of the shadow economy.
2. In the second step, the currency demand method is used to calibrate the relative SE estimates into absolute figures by using two or three values of the absolute size of the shadow economy from CDA estimations.

The following presented estimates are reached using the MIMIC and currency demand methods.^3^

## The effect of the Coronavirus pandemic on the shadow economy

The Coronavirus pandemic caused a severe recession in almost all European and OECD countries in 2020 and to a less extent also in 2021. The recession caused a strong rise in unemployment and a sharp decline of GDP and of national income. These major causal driving forces of the shadow economy had the effect of a strong increase in the shadow economies of these 36 countries. In Tables 1 to 3, the size and development of 31 European and of five non-European shadow economies are presented over the period 2003–2022.^4^ Let us first consider the results of the average size of the shadow economy of the 28 European Union countries over a more long-term perspective including the period before the Coronavirus pandemic occurred. In Table 1, one can see that the shadow economy in the year 2003 was 22.6% (of official GDP), which decreased to 19.6% in 2008 and increased to 20.1% in 2009 and then decreased again to 16.28% in 2019. Hence, we had in general a negative trend of the size of the shadow economies in almost all European and OECD countries. The main reason was the strong increase of GDP and an equally strong rise of national incomes. The results of these dynamics were much lower levels of engagement in shadow economy activities.

**Table 1:** Size of the shadow economy of the 27 EU countries + UK (up to 2020) over 2003–2022 (in % of off. GDP)

Country/year	2003	2004	2005	2006	2007	2008	2009	2010	2011	2012	2013	2014	2015	2016	2017	2018	2019	2020	2021	2022
Austria	10.8	11	10.3	9.7	9.4	8.1	8.5	8.2	7.9	7.6	7.5	7.8	8.2	7.8	7.1	6.72	6.1	7.23	6.9	7.05
Belgium	21.4	20.7	20.1	19.2	18.3	17.5	17.8	17.4	17.1	16.8	16.4	16.1	16.2	16.1	15.6	15.42	15.09	16.2	16.01	16.032
Bulgaria	35.9	35.3	34.4	34	32.7	32.1	32.5	32.6	32.3	31.9	31.2	31	30.6	30.2	29.6	30.84	30.12	32.93	32.41	33.05
Croatia	32.3	32.3	31.5	31.2	30.4	29.6	30.1	29.8	29.5	29	28.4	28	27.7	27.1	26.5	27.43	26.43	29.56	29.01	29.67
Czech Republic	19.5	19.1	18.5	18.1	17	16.6	16.9	16.7	16.4	16	15.5	15.3	15.1	14.9	14.1	13.61	13.07	14.22	13.92	13.48
Denmark	17.4	17.1	16.5	15.4	14.8	13.9	14.3	14	13.8	13.4	13	12.8	12	11.6	10.9	9.32	8.92	9.84	9.62	9.73
Estonia	30.7	30.8	30.2	29.6	29.5	29	29.6	29.3	28.6	28.2	27.6	27.1	26.2	25.4	24.6	23.21	22.06	23.59	23.06	22.74
Finland	17.6	17.2	16.6	15.3	14.5	13.8	14.2	14	13.7	13.3	13	12.9	12.4	12	11.5	11.02	10.59	11.36	10.94	10.83
France	14.7	14.3	13.8	12.4	11.8	11.1	11.6	11.3	11	10.8	9.9	10.8	12.3	12.6	12.8	12.52	12.37	13.59	13.12	14.21
Germany	16.7	15.7	15	14.5	13.9	13.5	14.3	13.5	12.7	12.5	12.1	11.6	11.2	10.8	10.4	9.74	8.54	10.42	10.03	8.81
Greece	28.2	28.1	27.6	26.2	25.1	24.3	25	25.4	24.3	24	23.6	23.3	22.4	22	21.5	20.81	19.23	20.94	20.31	20.93
Hungary	25	24.7	24.5	24.4	23.7	23	23.5	23.3	22.8	22.5	22.1	21.6	21.9	22.2	22.4	22.7	23.22	25.96	25.01	25.44
Ireland	15.4	15.2	14.8	13.4	12.7	12.2	13.1	13	12.8	12.7	12.2	11.8	11.3	10.8	10.4	9.7	8.91	9.86	9.4	10.13
Italy	26.1	25.2	24.4	23.2	22.3	21.4	22	21.8	21.2	21.6	21.1	20.8	20.6	20.2	19.8	19.51	18.66	20.42	20.15	20.32
Latvia	30.4	30	29.5	29	27.5	26.5	27.1	27.3	26.5	26.1	25.5	24.7	23.6	22.9	21.3	20.24	19.84	20.91	20.22	19.94
Lithuania	32	31.7	31.1	30.6	29.7	29.1	29.6	29.7	29	28.5	28	27.1	25.8	24.9	23.8	22.96	21.92	23.09	22.9	22.36
Luxembourg (Grand-Duché)	9.8	9.8	9.9	10	9.4	8.5	8.8	8.4	8.2	8.2	8	8.1	8.3	8.4	8.2	7.94	7.36	8.56	8.4	8.25
Malta	26.7	26.7	26.9	27.2	26.4	25.8	25.9	26	25.8	25.3	24.3	24	24.3	24	23.6	23.21	22.03	23.54	23.09	23.37
Netherlands	12.7	12.5	12	10.9	10.1	9.6	10.2	10	9.8	9.5	9.1	9.2	9	8.8	8.4	7.51	7.04	8.14	7.79	8.21
Poland	27.7	27.4	27.1	26.8	26	25.3	25.9	25.4	25	24.4	23.8	23.5	23.3	23	22.2	21.74	20.65	22.45	22.02	21.89
Portugal	22.2	21.7	21.2	20.1	19.2	18.7	19.5	19.2	19.4	19.4	19	18.7	17.6	17.2	16.6	16.13	15.38	17.01	16.5	15.71
Romania	33.6	32.5	32.2	31.4	30.2	29.4	29.4	29.8	29.6	29.1	28.4	28.1	28	27.6	26.3	26.66	26.9	29.33	28.89	29.03
Slovenia	26.7	26.5	26	25.8	24.7	24	24.6	24.3	24.1	23.6	23.1	23.5	23.3	23.1	22.4	22.16	21.54	23.07	22.49	22.08
South-Cyprus	28.7	28.3	28.1	27.9	26.5	26	26.5	26.2	26	25.6	25.2	25.7	24.8	24.2	23.6	23.21	22.08	24.32	23.65	23.9
Spain	22.2	21.9	21.3	20.2	19.3	18.4	19.5	19.4	19.2	19.2	18.6	18.5	18.2	17.9	17.2	16.61	15.36	17.39	16.9	15.81
Slovakia	18.4	18.2	17.6	17.3	16.8	16	16.8	16.4	16	15.5	15	14.6	14.1	13.7	13	12.83	12.15	14.01	13.66	13.06
Sweden	18.6	18.1	17.5	16.2	15.6	14.9	15.4	15	14.7	14.3	13.9	13.6	13.2	12.6	12.1	11.63	10.73	11.69	11.04	10.8
UK	12.2	12.3	12	11.1	10.6	10.1	10.9	10.7	10.5	10.1	9.7	9.6	9.4	9	9.4	9.8	9.56	10.67	10.19	10.93
28 EU countries/average (unweighted)	**22.6**	**22.3**	**21.8**	**21.1**	**20.3**	**19.6**	**20.1**	**19.9**	**19.6**	**19.3**	**18.8**	**18.6**	**18.3**	**17.9**	**17.3**	**16.8**	**16.28**	**17.87**	**17.42**	**17.29**

Source: Own calculations, as of January 2022. Remarks: The values for some countries in 2021 and for all in 2022 are projections. The UK left the EU on January 31, 2020. The so-called transition period ended on December 31, 2020

### Results in 2020

In 2020, the worldwide Coronavirus pandemic occurred and caused a severe recession in almost all countries. One consequence of this was a strong rise in the average size of the shadow economy to 17.87% (of GDP) of the 28 EU countries.^5^ Compared to 2019, this average increase is remarkably high at 1.59 percentage points (or by 9.8%) and is the highest seen over the last 20 years. In such a recession, a shrinking GDP and a strong increase of the unemployment rate are the key drivers of such a sharply rising shadow economy, as people try to compensate their official income loss with increased shadow economy activities.

**Table 2:** Size of the shadow economy of 3 non-EU European OECD countries over 2003–2022 (in % of off. GDP)

Country/year	2003	2004	2005	2006	2007	2008	2009	2010	2011	2012	2013	2014	2015	2016	2017	2018	2019	2020	2021	2022
Norway	18.6	18.2	17.6	16.1	15.4	14.7	15.3	15.1	14.8	14.2	13.6	13.1	13	12.6	12.2	11.8	10.8	11.62	11.05	10.4
Switzerland	9.5	9.4	9	8.5	8.2	7.9	8.3	8.1	7.8	7.6	7.1	6.9	6.5	6.2	6	5.8	5.5	6.07	5.82	5.61
Turkey	31.2	31.5	30.7	30.4	29.1	28.4	28.9	28.3	27.7	27.2	26.5	27.2	27	26.8	27.2	28.3	29.4	32.54	32.01	32.94
3 Non-EU countries/average	**20.1**	**19.7**	**19.1**	**18.3**	**17.6**	**17**	**17.5**	**17.2**	**16.8**	**16.3**	**15.7**	**15.7**	**15.5**	**15.2**	**15.1**	**15.3**	**15.2**	**16.74**	**16.29**	**16.32**
Unweighted average of all 31 European countries	**22.4**	**22.1**	**21.6**	**20.9**	**20.1**	**19.9**	**19.9**	**19.7**	**19.3**	**19**	**18.5**	**18.3**	**17.9**	**17.7**	**17.1**	**16.7**	**16.2**	**17.76**	**17.31**	**17.2**

Source: Own calculations, as of January 2022; values for some countries in 2021 and all in 2022 are projections on the basis of preliminary values

The strongest increase (by 3.13 percentage points) took place in Croatia, with a rise from 26.43% of official GDP in 2019 to 29.56% of GDP in 2020; the next strongest increase (2.81 percentage points) was seen in Bulgaria, where SE activities rose from 30.12% of GDP in 2019 to 32.93% of GDP in 2020. The weakest increase (at 0.77 percentage points) was found in Finland, where the SE rose from 10.59% of GDP in 2019 to 11.36% of GDP in 2020; the second lowest increase (0.92 percentage points) occurred in Denmark from 8.92% of GDP in 2019 to 9.84% of GDP in 2020.

### Results for 2021 and projected figures for 2022

With the help of projections for some countries, calculations can be made of the development of the shadow economies in European and OECD countries for 2021. In 2021, “only” a modest decrease of the of the shadow economy from 17.87% of GDP (2020) to 17.42% of GDP (2021; average value for the EU member countries) took place; hence, the average decline of the shadow economy of the EU countries will be 0.45 percentage points or 2.52%. The causes of this decline were massive public spending in infrastructure, subsidies to enterprises and special transfers to individuals which led to sizeable GDP growth combined with a decline in unemployment. The labor retention schemes applied in most OECD countries typically also partially replaced the previous labor income of workers which had effectively been laid off temporarily (see IMF 2021) — workers stayed on the payroll of the firm so that firm-specific human capital was effectively maintained despite lower aggregate demand, supply disruptions, and unanticipated liquidity constraints during the course of the pandemic (the effects of lockdowns, shutdowns, and increased uncertainty). In most cases, such workers had an excess of leisure time which they typically could use either in the self-service economy or in the shadow economy.

In 2022, a further recovery of the world economy will take place, but as the Coronavirus pandemic was to some extent still effecting most European and OECD countries in 2021 and will continue to do so even into 2022, a further the reduction of the shadow economy will be very modest. The average size of the shadow economy of the 28 EU member countries will decline from 17.42% (2021) to 17.29% in 2022. A decline will happen in 15 EU countries, while shadow economy activities will increase in 13 EU member countries.^6^ It should be noted that these are shadow economy predictions made in January 2022 where it is still open when the pandemic will be over and when the recovery of European and OECD countries will get stronger.

### Results for non-EU OECD countries

**Fig. 2:**
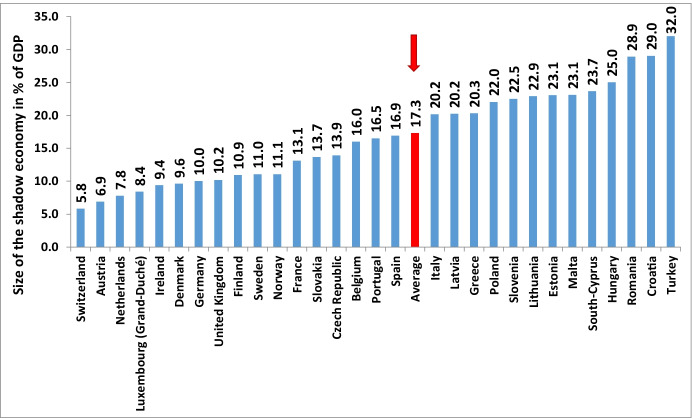
Size of the shadow economy of 31 European countries in 2021 (in % of off. GDP). Source: Own calculations, as of January 2022

Turning to the development of the shadow economy in three non-EU but European OECD countries, the results are shown for the period from 2003 to 2022 in Table 2. As was the case for the 28 EU countries, the shadow economy of these three non-EU European countries sharply increased from 2019 to 2020: in Norway from 10.8 to 11.62% of GDP, in Switzerland from 5.5 to 6.1%, and in Turkey from 29.40 to 32.54%. The strongest increase (at 3.14 percentage points or 10.4%) occurred in Turkey. Due to a quite strong economic recovery in 2021 and in 2022, one can predict a small decrease of the shadow economy — roughly by 0.42 percentage points — in these three countries. If we combine these three European countries and the 28 EU countries, the average size of the shadow economy of the 31 European countries was 22.4% in 2003, decreased to 19.4% in 2008, then increased to 19.9% in 2009, and again decreased to 16.20% in 2019. It increased sharply to 17.76% in 2020 and will decline to 17.20% in 2022 (see Table 2, but also compare Fig. 2 (values for 2020) and 3 (values for 2021)).

Next, we turn to the development of the shadow economy of the highly developed Non-European OECD countries, namely Australia, Canada, Japan, New Zealand, and the USA over the period from 2003 to 2022; results are presented in Table 3. As with the 31 European countries, calculations show a strong increase of the shadow economy of these 5 countries from 7.6% (the average value of 2019 GDP) to 8.6% of GDP in 2020: a rise of 1.0 percentage point or 13% (for more details, see Table 3). In 2021 and 2022, one can forecast a modest decline in the shadow economies of these five countries by 0.3 percentage points, as an economic upswing took place in 2021 and is expected to continue in all five countries.

**Table 3:** Size of the shadow economy of 5 highly developed non-European OECD countries over 2003–2022 (in % of off. GDP)

Country/year	2003	2004	2005	2006	2007	2008	2009	2010	2011	2012	2013	2014	2015	2016	2017	2018	2019	2020	2021	2022
Australia	13.7	13.2	12.6	11.4	11.7	10.6	10.9	10.3	10.1	9.8	9.4	10.2	10.3	9.8	9.4	9.2	8.9	9.71	9.52	9.31
Canada	15.3	15.1	14.3	13.2	12.6	12	12.6	12.2	11.9	11.5	10.8	10.4	10.3	10	9.8	9.6	9.4	10.32	9.74	9.95
Japan	11	10.7	10.3	9.4	9	8.8	9.5	9.2	9	8.8	8.1	8.2	8.4	8.5	8.6	8.5	8.2	9.1	8.84	8.56
New Zealand	12.3	12.2	11.7	10.4	9.8	9.4	9.9	9.6	9.3	8.8	8	7.8	8	7.8	7.4	6.9	6.8	7.86	7.25	7.63
USA	8.5	8.4	8.2	7.5	7.2	7	7.6	7.2	7	7	6.6	6.3	5.9	5.6	5.4	5.1	4.8	6.11	6.56	6.1
Other OECD countries/unweighted average	12.2	11.9	11.4	10.4	10.1	9.6	10.1	9.7	9.5	9.2	8.6	8.6	8.6	8.3	8.1	7.9	7.6	8.6	8.4	8.31

Source: Own calculations, as of January 2022; values for some countries in 2021 and for all in 2022 are projections on the basis of preliminary values

Finally, we consider the development of the shadow economy of all 36 European and OECD countries over the period from 2003 to 2022; the various averages are shown in Table 4. Again, one can see that the average size of the shadow economy of all 36 European and OECD countries strongly increases from 14.98% of GDP in 2019 to 16.48% of GDP in 2020 (a rise of 1.5 percentage points or 11%). Due to a strong economic recovery, the shadow economy slightly decreased to 16.07% of GDP (the average of all 36 countries) in 2021 and will further decrease, albeit modestly, to 15.96% of GDP in 2022. As mentioned previously,^7^ the most important reason for this decrease is that if the official economy is recovering or booming, people have fewer incentives to undertake additional activities in the shadow economy and to earn extra clandestine money. The decrease is stronger in those countries where corruption is low or good governance is in place.

**Table 4:** Size of the shadow economy of 36 European and OECD countries of various unweighted averages over 2003–2022 (in % of off. GDP)

Averages/year	2003	2004	2005	2006	2007	2008	2009	2010	2011	2012	2013	2014	2015	2016	2017	2018	2019	2020	2021	2022
28 EU countries/average (unweighted)	22.60	22.30	21.80	21.10	20.30	19.60	20.10	19.90	19.60	19.30	18.80	18.60	18.30	17.90	17.30	16.80	16.28	17.87	17.42	17.29
3 Non-EU countries/average (unweighted)	20.10	19.70	19.10	18.30	17.60	17.00	17.50	17.20	16.80	16.30	15.70	15.70	15.80	16.00	15.10	15.30	15.20	16.74	16.29	16.32
5 Other OECD countries/average (unweighted)	12.20	11.90	11.40	10.40	10.10	9.60	10.10	9.70	9.50	9.18	8.60	8.60	8.60	8.30	8.10	7.90	7.61	8.60	8.40	8.31
All 36 countries/average (unweighted)	21.00	20.70	19.40	19.40	18.70	18.00	18.50	18.30	18.00	17.60	17.10	17.00	16.70	16.40	15.80	15.40	14.98	16.48	16.07	15.96

Source: Own calculations, as of January 2022; values for some countries in 2021 and for all in 2022 are projections on the basis of preliminary values

## Summary and policy conclusions

**Fig. 3:**
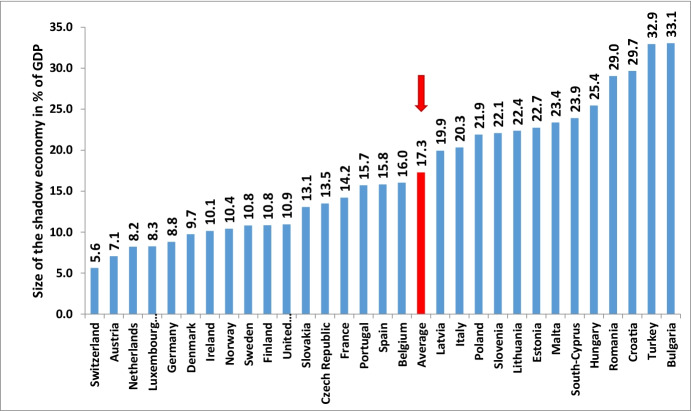
Size of the shadow economy of 31 European countries in 2022 (in % of off. GDP). Source: Own calculations, as of January 2022; all values are projections

To summarize, there are four different developments with respect to the dynamics of the shadow economy of these 36 European and OECD countries up to 2022:

1. In 2020, a strong increase of the shadow economy from 14.98% of GDP (2019) to 16.48% of GDP (2020) is observed; i.e., a 1.5 percentage points or 10% increase — the strongest increase of the average figure over the last 20 years. The main reason of this increase is the worldwide Coronavirus pandemic and the resulting severe recession which affected most countries. For 2022, a modest decline of the shadow economy — by roughly 0.52 percentage points — is forecasted. The main reason being the recovery of the official economy in 2021 and the forecasted continued recovery in 2022 (as of January 2022).
2. Eastern, Central, and Southern European countries, such as Bulgaria, Cyprus, Czechia, Latvia, Lithuania, and Poland, have higher shadow economies in comparison to the “old” western European Union countries, such as Austria, Belgium, Germany and Italy. Hence, we have an increase of the size of the shadow economy from west to east.
3. In addition, an increase in the size and development of the shadow economy can be observed from north to south. On average, the Southern European countries have considerably higher shadow economies than those of Central and Western Europe. Figures 2 and 3 also demonstrate both movements.
4. The five non-European highly developed OECD countries (Australia, Canada, Japan, New Zealand, and the USA) have lower shadow economies with an average size of about 8.40% of GDP in 2021 and predicted one of 8.3% of GDP in 2022, which is lower than the SE estimates for most European countries.

Finally, one can make the following four policy conclusions:

1. Since 2020, and in every country, the challenge for all governments was to undertake policy measures aimed at stimulating the official economy with strong GDP growth and a reduction of unemployment rates in order to reduce the shadow economy. The more successful such policy measures are, the more the shadow economy declines. Most countries have indeed enjoyed some success..
2. However, the crucial question remains: “Is this reduction of the shadow economy a blessing or a curse?”

My answer is:

(i) If one assumes that roughly 50% of all shadow economy activities complement those of the official sector (i.e., those goods and services would otherwise not be produced in the official sector), the development of the total (official + shadow economy) GDP is always higher than the “pure” official one.
(ii) A decline of the shadow economy will only increase the total welfare in a country if policymakers succeed in transferring economic activity from the shadow economy into the official economy.
(iii) Therefore, policymakers have to favor and choose such policy measures as strongly increase the incentives to transfer the production from the shadow (black) to the official sector.

Hence, the conclusion of these two remarks is: The decline of the shadow economy will only be a blessing for the economy as a whole, if incentive-orientated policy measures will be applied, which I strongly recommend.

Considering the latest developments during the fall of 2021 and from February 2022, I add two further policy conclusions:

(3) The massive recessions occurring in most European and OECD countries in the pandemic went along with high government deficits and rising expected long run public debt-GDP ratios. This could generate medium-term pressure in many countries to raise tax rates which in turn would make it difficult to reduce the size of the shadow economy.
(4) As regards the Ukraine-Russia war which started in February 2022, many European and OECD countries will face a large wave of refugees who will face initial impediments to working in the official economy. Since standard modeling of immigration and refugee waves suggests that Eastern European countries would receive the highest shares of refugees from Ukraine, the lead position of Eastern European EU countries in terms of the size of the shadow economy in the overall EU will be reinforced in the medium term.

## Data Availability

The datasets generated during and/or analyzed during the current study are available from the corresponding author on reasonable request.

## Footnotes

1 For recent surveys, compare Feld and Schneider (2010), Medina and Schneider (2021), and Williams and Schneider (2016). For debates and controversies compare Kirchgaessner (2016) and Feld and Schneider (2016), Breusch (2016), Feige (2016a, 2016b), Schneider (2016), and Hashimzade and Heady (2016).

2 As many papers exist which extensively present the MIMIC method with all its strengths and weaknesses, a detailed presentation is not done here. Compare, e.g., Dell’Anno (2021a, 2021b), Medina and Schneider (2021), and Dybka et al. (2019).

3 In Medina and Schneider (2018, 2021), econometric estimations are shown from which the size of the shadow economy can be calculated.

4 As explained in Sect. 1, the calculation of the size and development of the shadow economy is done by applying the MIMIC (Multiple Indicators and Multiple Causes) estimation procedure. Using the MIMIC estimation procedure, one gets only relative values, and one needs other methods such as the currency demand approach or the income discrepancy method in order to calibrate MIMIC values into absolute values.

5 The calculated values for 2021 are still projections for some countries; for 2022, they are projections for all countries based on the forecasts of the official figures (GDP, unemployment, etc.) of these countries. I am aware that the UK officially left the European Union on January 31, 2020, but due to the long membership of the UK in the EU, I did not change the categorization of the UK from Table 1 to Table 2.

6 As these values are still projections and as the Coronavirus pandemic is not yet over in early 2022, an in-depth discussion and comparison of the shadow economy for individual countries is not done.

7 For a detailed discussion of the importance of the development of the official economy on the shadow economy, see see Feld and Schneider (2010), Alm and Embaye (2013), Abdih and Medina (2013), and Medina and Schneider (2021).
